# Conflicting identities and cooperation between groups: experimental evidence from a mentoring programme

**DOI:** 10.1098/rspb.2025.1363

**Published:** 2025-08-06

**Authors:** Antonio M. Espín, Maria Paz Espinosa, Maria J. Vázquez-De Francisco, Pablo Brañas-Garza

**Affiliations:** ^1^ Department of Applied Economics, Universidad de Granada, Granada, Andalucia, Spain; ^2^ Department of Economics, University of the Basque Country, Bilbao, Basque Country, Spain; ^3^ ETEA Foundation, Development Institute, Universidad Loyola Andalucia, Cordoba, Andalucía, Spain; ^4^ Loyola Behavioral Lab, Universidad Loyola Andalucia, Cordoba, Andalucía, Spain

**Keywords:** group diversity, natural identities, negative stereotypes, social integration, stigmatization, outgroup favouritism

## Abstract

Well-functioning human societies require the integration of vulnerable minorities, yet leading scientific theories conflict on how easily diverse groups cooperate. We experimentally investigate cooperation in 14 centres of a mentoring programme where participants have two possible natural identities—individuals raised under legal guardianship, suffering a negative stereotype (*G*; *n =* 112) and users without such a social stigma (*NG*; *n =* 82). Participants played a prisoners’ dilemma game with an anonymous partner from the same centre (centre-ingroup) and from another centre (centre-outgroup). For individuals without a history within-centre interaction, we find centre-outgroup favouritism among *G* and centre-ingroup favouritism among *NG*. However, the longer *G* individuals have been in the centre the more centre-ingroup favouritism they display, while the opposite is true for *NG*. Regardless of within-centre history, both *G* and *NG* individuals cooperate less with the centre-ingroup (versus outgroup) as the probability that the centre-ingroup is *G* increases. Thus, we observe patterns of centre-outgroup and natural-outgroup favouritism among *G* which challenge theoretical frameworks exclusively focusing on ingroup favouritism. Our findings highlight the roles of system-justification and stereotypes in intergroup cooperation and have implications for the integration of vulnerable groups and the optimization of social policy programmes.

## 1. Introduction

Interactions among individuals with different group identities are ubiquitous in human societies [1–4]. Social integration and acceptance refer to the relationship between an individual and the social environment and are usually measured through involvement in the community [5,6]. The integration of minorities or special groups in a society are key for its success, but it dramatically depends on how these groups are perceived by the population at large. From an economic perspective, it is well-known that the absence of social integration may generate inefficiencies [7,8]. Hence, modern societies face the integration of migrants and minorities as a major issue.

In this paper, we study social integration between two natural groups participating in a mentoring programme. The programme aims at expanding employment opportunities for young adults at risk of social exclusion in Andalusia, Spain. A relevant feature of the programme is that it deals with individuals raised in foster homes (under legal guardianship: group *G*) and other young users (group *NG*)*.*^1^ The interaction between these two groups of users is a fundamental concern of the programme leaders. *G* members are negatively stereotyped in society (e.g. as more likely to be involved in illegal activities, abuse drugs, etc. [10–12]), and suffer the stigma of belonging to that group, whereas *NG* do not. This sets a strong natural-identity distinction between the two groups, with one of them (*G*) being clearly disadvantaged as compared to the other.

People often identify with their social group and take actions that benefit the ingroup compared to the outgroup [13]. One frequently observed pattern is therefore ingroup favouritism (stemming from ‘ingroup love’ and/or ‘outgroup hate’ [14,15]). Such ‘parochial altruism’ is seen as responsible for the existence of conflicts between human groups [16,17]. Moreover, even minimal groups based on trivial categorizations (e.g. art preferences or test scores) can trigger group identification and ingroup favouritism; a finding that gave rise to the so-called social identity theories.

To explain ingroup favouritism, two leading theoretical perspectives are those of social identity theory [18,19] and group-bounded generalized reciprocity [20]. Broadly, the former explains ingroup favouritism as a result of group categorization; the mere (self-)categorization as a member of a particular group leads individuals to favour their ingroup, as a means of maintaining a positive social identity and favourable social comparisons. The latter, on the other hand, explains ingroup favouritism using an evolutionary lens, as stemming from the existence of an exchange system based on indirect reciprocity restricted to the boundaries of the ingroup, and where the heuristic of expected reciprocation by others within the system is the key underlying force of ingroup cooperation. However, especially among members of disadvantaged groups, patterns of outgroup favouritism are often observed, but these observations have typically remained outside the debate between these two theoretical traditions which focus on ingroup favouritism [21].

In contrast, the theoretical frameworks built upon system-justification and stereotypes [21,22] place more emphasis on the possibility of outgroup favouritism and highlight that some individuals may not regard their own group positively [23–27] . This peculiarity has been typically observed in disadvantaged or negatively stereotyped individuals who even often disfavour their own group through internalized inferiority, thus ‘justifying’ and reinforcing the status quo stratification of the social system as a strategy to psychologically coping with one’s own state of disadvantage. Examples in the literature abound (see [3] for the punishment of uncooperative group members among Spanish Romani people, and [28] for the sharing behaviour of minority East Asian children in Canada).

The empirical analysis of between-group interaction is further compounded by the fact that people have multiple social/group identities, with one or the other being prompted by different circumstances [18,29,30]. Often, it is the presence of the outgroup that makes a particular identity salient and alters social interaction norms—for example, a national or ethnic identity can be salient in encounters with people from other countries or ethnicities [3,31]—although in some cases, people can also strategically emphasize the identities that can be more contextually beneficial [29]. This important aspect is the main focus of our study: the presence of a double identity and its effects on cooperation and social integration.

The traditional treatment of group identity in the literature has been limited to single ingroup-outgroup categorizations. Yet, a few studies have recently started to analyse how more realistic multiple cross-categorizations affect social behaviour by pairing subjects explicitly sharing none, one or two (out of two) group identities. Kumar *et al*. [32] show that nationality is more important than gender categorization for ingroup favouritism in cooperation, indicating that some group identities dominate others when considered together. In contrast, Hong *et al*. [33] and Uğurlar *et al*. [34] find that favouritism increases with the number of identities shared with the recipient, regardless of whether the two dimensions are (nearly) minimal, or based on political orientation or religious affiliation. Imada *et al*. [35] also find such an additive impact of the two dimensions (both minimal) on cooperation and indicate that expected cooperation by the other party is the main driver of ingroup favouritism. Finally, the results of [36] qualify the above findings by suggesting that it is only some individuals (labelled as ‘groupy’) who favour their ingroup across different categorizations, in this case, a minimal and a real political-leaning-based grouping.

We contribute to this emerging literature by analysing how multiple group identity affects cooperation in presence of strong natural identities with different status [37]. We address this in a lab-in-the-field experiment conducted within a mentoring programme where two strong natural identities coexist: members of groups *G* (individuals raised under legal guardianship, suffering a negative stereotype) and *NG* (users without such a social stigma). Additionally, we consider a second dimension given by belonging to a particular training centre (a purely administrative categorization). The final sample consists of 192 participants (112 *G*, 82 *NG*) from 14 centres. See §4.

Participants played a Prisoner's Dilemma (PD) with two anonymous counterparts (random order), yielding our second identity dimension: one from the same centre (centre-ingroup) and another from another centre (centre-outgroup). Decisions were incentivized such that participants earned lottery tickets for monetary prizes.

This experimental design makes centre-based identity salient. Since each centre has a mix of the two natural identities among their participants (*G*/*NG*), which is salient by itself owing to the importance of this distinction within the programme, our design allows us to explore how the *G*/*NG* composition affects cooperation along the second dimension. During the experiment in each centre, all participants were in a single room and could see each other and therefore guess the session composition regarding natural identities. We define the variable ‘similarity’ as the proportion of subjects with the same identity as the decision-maker participating in her centre (see [38] for a related similarity measure based on religiosity).

Similarity is *exogenous* and is determined by attendance at each session, entailing that it is specific to each subject and is only shared by subjects with the same identity in the same centre. For those individuals not knowing the identity status of others for any reason, other sources of information can be used to infer the session composition, in particular, age and nationality are two such potential sources (*G* individuals are younger and more likely to be non-Spanish than *NG*; see §2).

In sum, we exploit between-subjects variation in similarity with the centre-ingroup and within-subjects variation in cooperation with the centre-ingroup versus centre-outgroup. Note that, participants cannot compute similarity with the centre-outgroup in any way, as they were never informed what centre the outgroup belonged to. While our design leads to losing control over certain variables compared to designs in which both dimensions are experimentally manipulated, it is also more ecologically realistic than previous experiments in several aspects. First, we do not make the natural-identity distinction explicit, thus reducing potential demand effects [39] in that regard and making the context more realistic, as in real life identity inferences are frequently intuitive, not explicit. Second, in everyday contexts, people often perceive outgroups as homogenous and defined by a single identity, whereas ingroups are seen as more complex and multifaceted; the closer the group, the more likely individuals are to notice and consider internal differences and nuanced traits [40]. Thus, that the natural-identity distinction only operates for the centre-ingroup seems also realistic.

By definition, individuals’ cooperation along these two identity dimensions in the PD game could yield the following cases (excluding the possibility of no effect for brevity):

— Favouritism towards the centre-ingroup versus outgroup.
— Favouritism towards the natural-ingroup reflected as a positive effect of similarity on centre-ingroup favouritism.
— Favouritism towards the centre-outgroup versus ingroup.
— Favouritism towards the natural-outgroup reflected as a negative effect of similarity on centre-ingroup favouritism.

We approach the problem using a simple generalized model of other-regarding preferences with social identity concerns, a group-contingent social preference model [41]. In particular, individuals in our framework attach a subjective value to the welfare of an interaction partner based on identity considerations. This framework allows us to put forward a series of predictions with solid game-theoretical foundations (see §4).

In the case of the centre-based grouping, we test whether individuals cooperate more with their own centre on average (prediction A1: ‘*centre-ingroup favouritism*’) or not; i.e. we test the existence of centre-ingroup or centre-outgroup favouritism. For the natural identities, we test whether the effect of similarity is positive or negative, yielding three mutually exclusive predictions:^2^

— B1. ‘*Natural-ingroup favouritism*’: similarity increases centre-ingroup favouritism for both *G* and *NG*.
— B2. ‘*Stigma of G*’: similarity increases centre-ingroup favouritism among *NG* but decreases centre-ingroup favouritism among *G*.
— B3. ‘*Sympathy towards the disadvantaged G*’: similarity increases centre-ingroup favouritism among *G* but decreases centre-ingroup favouritism among *NG*.

Finally, people’s preferences can also evolve with interaction. Specifically, the training centres allow these young individuals to meet new people and provide them with new skills. Therefore, the time each participant spends in the centre may be relevant. Our data allow testing the impact of the length of interaction *history* with the centre-ingroup, as we specifically sampled participants in different stages of the programme.

Most importantly, we can study whether the centre-based identity reinforces over time and eventually overcomes the effect of natural identity. In other words, repeated interaction over time within the centre might increase the perception of belonging and interdependence, but it might also trigger negative feelings towards ingroups or outgroups, affecting the behavioural dynamics in complex ways [1,16,20,42].

For the newcomers, the situation shares some elements of the standard minimal group paradigm because grouping responds to meaningless administrative reasons and there is no previous interaction; depending on the definition used, the centre can also be considered a ‘weak real identity’ for newcomers [43]. For participants with a long interaction history, however, our centre-based grouping is clearly more meaningful. In this vein, interaction history within the same centre could reduce the barriers between groups *G* and *NG* and promote social integration by blurring the effect of natural identities, i.e. of similarity with the centre-ingroup (see §4).

In sum, our data analysis explores the likelihood of cooperating with the centre-ingroup/outgroup counterparts for both *G* and *NG* individuals and how this is modulated by both similarity and interaction history with the centre-ingroup in order to test our predictions. The results can be rationalized using our theoretical framework, supporting the prediction B2, *stigma of G individuals*. Interaction history increases centre-ingroup favouritism among *G* individuals and reduces it among *NG* individuals, yet it never overcomes the effect of natural identities.

Our results contribute to the experimental literature on the effects of natural identities on intergroup behaviour in economic games with non-standard samples [2–4,44–49]. These experiments pinpoint the complexity of combining natural groups with different status/entitativity and the role of stereotypes and prejudice for intergroup-contact outcomes, claiming for more research and theoretical accounts that consider all these factors, also in multi-identity situations.

## 2. Results

We focus on the likelihood of cooperating with the centre-ingroup as compared to the likelihood of cooperating with the centre-outgroup (see §4). This is our main dependent variable, labelled ‘centre-ingroup favouritism’; it can take values −1, 0 or 1 (indicating centre-outgroup favouritism when negative).

### (a) Descriptive results

#### (i) Centre grouping

Contrary to the standard result [13,50,51], we observe no centre-ingroup favouritism on average. The mean (±s.e.m. clustered on enumerators) centre-ingroup favouritism is 0.005 ± 0.045, which is statistically undistinguishable from zero (*p* = 0.90; Wilcoxon signed-rank test). While 68% of subjects make the same choice in the PD (i.e. cooperate or defect) for the centre-ingroup versus the centre-outgroup counterpart, 16% of them cooperate more with the centre-ingroup, and another 16% cooperate more with the centre-outgroup.

Moreover, we see that *G* and *NG* participants are equally likely to show no effect of centre identity (the percentage showing no differences between centre-ingroup and centre-outgroup cooperation is similar: 71% versus 62%, *p* = 0.22; Fisher’s exact test). In both cases, the proportion of individuals with centre-ingroup or centre-outgroup favouritism is nearly identical (15% and 13% for *G*; 18% and 20% for *NG*). As a result, both types display average centre-ingroup favouritism levels which are undistinguishable from zero (0.018 ± 0.037 for *G*, −0.012 ± 0.085 for *NG*, both *p* > 0.72; Wilcoxon signed-rank test) and from each other (*p* = 0.72; Mann–Whitney rank-sum test).

*Result 1:* We find no centre-ingroup favouritism on average for either *G* or *NG* individuals.

These results go against the standard ingroup bias and therefore, prediction A1 does not hold overall. In what follows we delve into the role of similarity.

#### (ii) Similarity in natural identities

**Figure 1.**
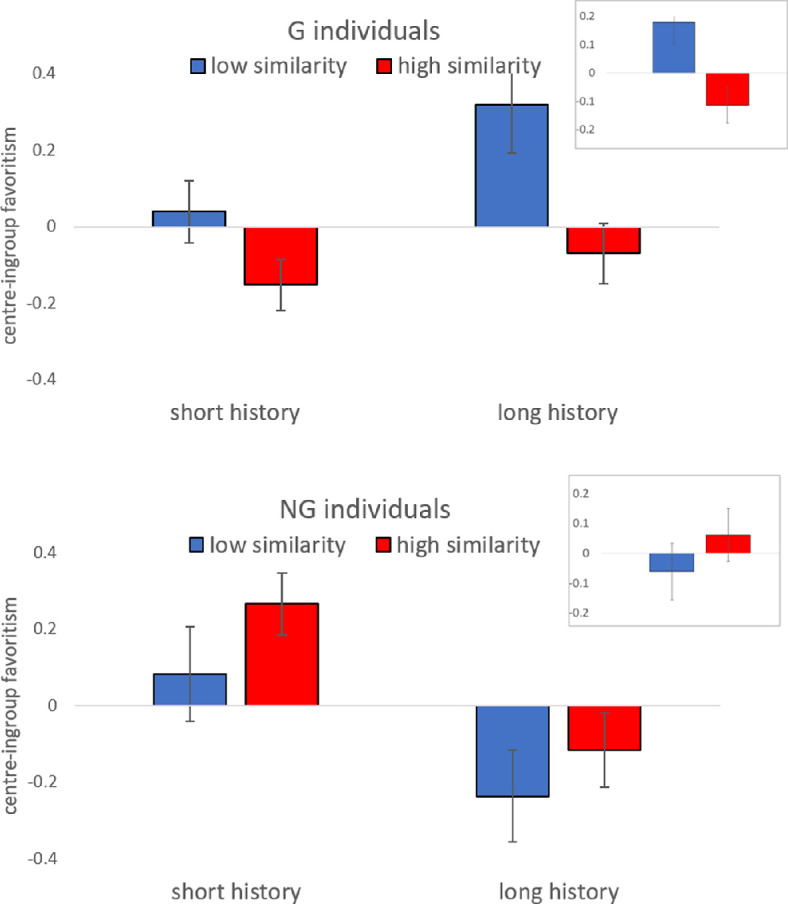
Average centre-ingroup favouritism for below- and above-median centre-ingroup similarity (low, in blue versus high, in red) and interaction history (short versus long). Top panel: *G* subjects. Bottom panel: *NG* subjects. The top-right small embedded figures depict the similarity breakdown without considering interaction history. Error bars represent s.e.m. clustered on enumerators.

In contrast to the above null findings on aggregate centre-ingroup favouritism, we do observe large discrepancies between *G* and *NG* individuals when we break down the sample into two groups of similarity, namely, below-median and above-median (i.e. similarity = 0.52). These comparisons are plotted on the top-right embedded figures of figure 1 (top panel for *G*, bottom panel for *NG*). For *G* individuals, there is a decline in centre-ingroup favouritism when we move from below- to above-median similarity, whereas the opposite is observed for *NG*. This result would support prediction B2 (i.e. *stigma of group G*) against B1 and B3.

#### (iii) Interaction history

The main plots in figure 1 show the mean centre-ingroup favouritism for below- and above-median similarity, breaking down by below- and above-median interaction history (median history = 35 weeks). The figure suggests opposite effects of interaction history on centre-ingroup favouritism for *G* (positive effect) and *NG* subjects (negative effect).

### (b) Main regression analysis

In this section, we test the significance of the above preliminary evidence using regression analysis. Instead of using the median split, the regressors similarity and history are defined as continuous variables. The regressions also allow us to control for other potential confounding factors.

Table 1 reports a series of GLMM regression models with random intercepts at enumerators, estimating centre-ingroup favouritism as a function of decision maker’s natural identity (*G* versus *NG*), interaction history (from 0 to 74 weeks), and similarity within the centre-ingroup (from 0 to 1). This GLMM specification allows us to control for the potential levelling effects of enumerators on centre-ingroup favouritism.

**Table 1.** Centre-ingroup favouritism as a function of natural identity *G*/*NG*, and centre-ingroup history and similarity. Notes: GLMM estimates of centre-ingroup favouritism as a function of *G* versus *NG* status, history and similarity. Standard errors in parentheses. *p < 0.10, **p < 0.05, ***p < 0.01. For significant critical estimates in columns (2) and (6), *p*-values adjusted for multiple hypotheses testing using the Holm [52] method are presented in square brackets.

centre-ingroup favouritism	(1)	(2)	(3)	(4)	(5)	(6)	(7)	(8)
*G* (versus *NG*)	0.055	0.193	0.181	−0.128	0.006	0.135	0.121	−0.200
	(0.086)	(0.236)	(0.242)	(0.378)	(0.111)	(0.233)	(0.240)	(0.372)
history (weeks)	0.001	−0.007***	−0.006	−0.010*	0.002	−0.006**	−0.005	−0.009
	(0.002)	(0.003) [0.016]	(0.005)	(0.006)	(0.002)	(0.003) [0.044]	(0.005)	(0.006)
similarity	−0.149	0.491**	0.543	0.277	−0.145	0.483*	0.543	0.258
	(0.175)	(0.246) [0.046]	(0.353)	(0.432)	(0.177)	(0.249) [0.052]	(0.352)	(0.432)
*G* × history		0.012***	0.013***	0.022**		0.013***	0.013***	0.023**
		(0.003) [0.001]	(0.004)	(0.009)		(0.003) [0.001]	(0.004)	(0.009)
*G* × similarity		−1.009***	−1.008***	−0.467		−0.961***	−0.960***	−0.383
		(0.337) [0.009]	(0.337)	(0.610)		(0.334) [0.012]	(0.334)	(0.611)
history × similarity			−0.002	0.006			−0.002	0.006
			(0.007)	(0.010)			(0.007)	(0.010)
*G* × history × similarity				−0.015				−0.016
				(0.014)				(0.014)
order (ingroup first)					0.151*	0.179**	0.179**	0.181**
					(0.081)	(0.078)	(0.078)	(0.078)
female					−0.184*	−0.137	−0.137	−0.133
					(0.102)	(0.098)	(0.098)	(0.097)
age					−0.004	−0.001	−0.001	0.001
					(0.012)	(0.012)	(0.012)	(0.012)
Spanish					0.080	0.089	0.088	0.097
					(0.103)	(0.099)	(0.099)	(0.099)
constant	0.026	−0.033	−0.058	0.065	0.052	−0.124	−0.151	−0.069
	(0.126)	(0.150)	(0.190)	(0.222)	(0.306)	(0.295)	(0.316)	(0.324)
*χ* ^*2*^	1.031	19.881***	19.928***	21.176***	8.094	28.492***	28.560***	30.016***
Ll	−165.633	−156.705	−156.683	−156.119	−162.164	−152.876	−152.846	−152.213
*n*	194	194	194	194	194	194	194	194

In column (1), the main effects of the three explanatory variables are not significant (*p* > 0.39; see effect sizes reported as linear coefficients in the table). In column (2), we introduce the interactions between the decision maker’s natural identity and the variables history and similarity. As expected from figure 1, both interaction effects are significant (*p* < 0.01): similarity matters for group *NG* with a positive effect (0.491, *p =* 0.046), while it is negative for group *G* and of about the same size (−0.518, *p* = 0.025). The difference in absolute value between the two coefficients is not significant (*p* = 0.94; Wald test). This result points to the stigma prediction B2: *all players are more willing to favour NG than G individuals*. In addition, interaction history has a negative effect on centre-ingroup favouritism for *NG* individuals (−0.007*, p =* 0.008) and a positive effect for *G* individuals (0.006*, p =* 0.014). These effects are again of similar magnitude (*p* = 0.74; Wald test). This points to *G* individuals feeling more integrated in the centre over time, and the opposite for those of *NG*.

Finally, in columns (3) and (4), the interaction between similarity and history and the three-way interaction with decision maker’s natural identity do not yield significant effects (*p* > 0.28). This means that the effects of similarity and history are independent.

In sum, we find opposite and almost identically strong effects of both similarity and interaction history on centre-ingroup favouritism for *G* and *NG*. In columns (5)–(8), we include controls for decision order (PD played first with the centre-ingroup or the centre-outgroup), gender, age and nationality (Spanish or not). These are potential confounding factors of any difference between *G* and *NG* because *G* individuals are younger (mean ± s.e.m. age: *G* = 20.36 ± 0.25, *NG* = 24.86 ± 0.51) as well as more likely to be non-Spanish (mean ± s.e.m. proportion of Spanish: *G* = 0.32 ± 0.04, *NG* = 0.85 ± 0.04) and males (*G =* 0.85 ± 0.03*, NG* = 0.46 ± 0.06) than *NG* (all *p* < 0.01; OLS). The main results remain qualitatively similar after introducing these controls. We summarize our findings as follows:

*Result 2:* Similarity affects centre-ingroup favouritism negatively for *G* individuals and positively for *NG* individuals.

*Result 3:* History affects centre-ingroup favouritism positively for *G* individuals and negatively for *NG* individuals.

*Result 4:* The effects of similarity and history are independent.

The data support the stigma prediction B2: there is discrimination against the negatively stereotyped group *G* (result 2), while there is no centre-ingroup favouritism on average (i.e. prediction A1 does not hold; result 1). The effect of history (result 3) suggests that repeated interaction increases positive regard for the centre-ingroup among *G* and the opposite among *NG*. Yet, the impact of similarity is not affected by history (or vice versa; result 4).

**Figure 2.**
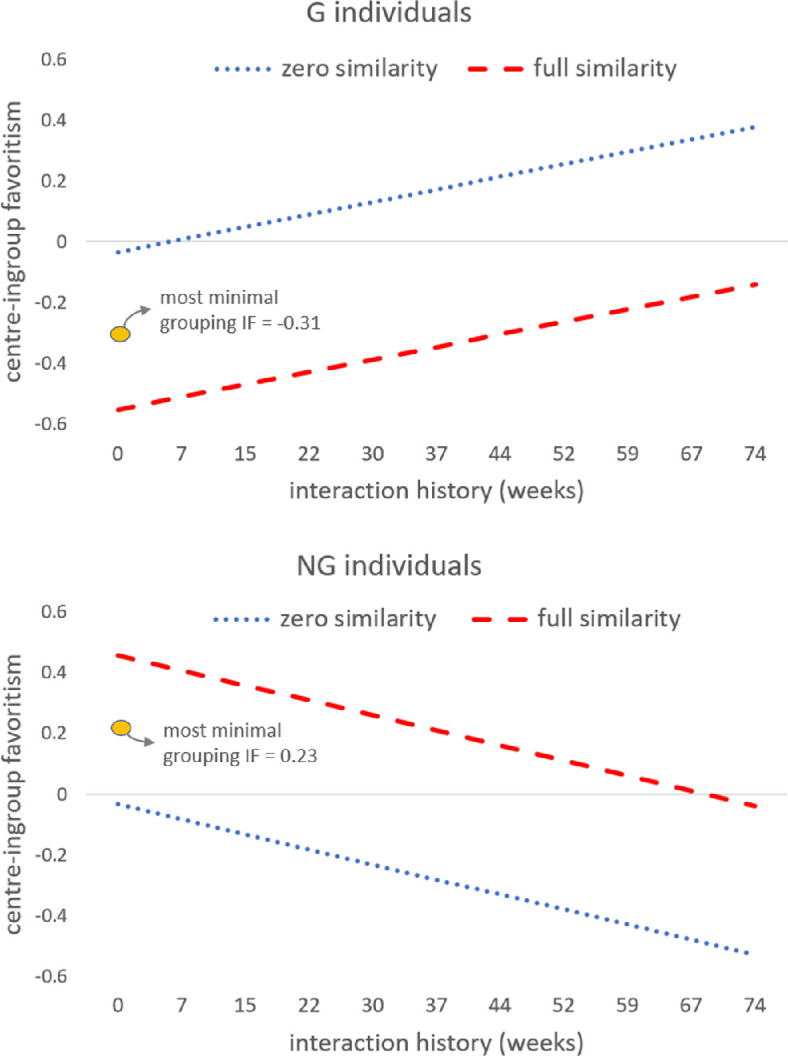
Centre-ingroup favouritism as a function of similarity and interaction history. Top panel: *G* individuals. Bottom panel: *NG* individuals. GLMM estimates from column (2) of table 1. For visual clarity, we plot the linear effect of interaction history on centre-ingroup favouritism evaluated at similarity = 0 (dotted blue line) and similarity = 1 (dashed red line). The average centre-ingroup favouritism (IF) estimated at zero interaction history (‘most minimal’ grouping) is reported as an orange circle.

Figure 2 displays the centre-ingroup favouritism estimated in column (2) of table 1. The top and bottom panels refer to *G* and NG individuals, respectively. We plot the estimated effect of interaction history on centre-ingroup favouritism at the two extreme cases of similarity, that is, for zero and *full similarity* (zero [full] similarity means that all other participants in the centre are of different [the same] natural identity as the decision maker). An orange circle depicts the centre-ingroup favouritism estimated for the case of zero interaction history (i.e. newcomers), at the average level of similarity for *G* and *NG*. This provides an estimation of the average centre-ingroup favouritism for the ‘most minimal’, or weakest, centre identity. We find centre-outgroup favouritism for *G* (−0.313, 95%CI = [−0.544, −0.082]) and centre-ingroup favouritism for *NG* (0.232, 95%CI = [0.015, 0.448]).

An interesting pattern is that in those centres with a larger proportion of *G* participants, the level of centre-ingroup favouritism is smaller (coeff *=* −0.344, *p =* 0.037, in a GLMM regression with proportion of *G* participants in the centre as explanatory variable). Note that, this variable is different from similarity as it only considers the proportion of *G*/*NG* participants in the centre, not the probability of being matched with someone of the same type. This result holds even though *G* individuals are not less cooperative than those of *NG* with the centre-ingroup (*p* = 0.38) nor with the centre-outgroup (*p* = 0.19; Fisher’s exact test). This finding is related to the stigma prediction and reflects a self-fulfilling prophecy, coming from the aggregation of negative stereotypes [53,54]. Since both *G* and *NG* disfavour *G* individuals, then the proportion of *G* participants in a centre reduces the level of cooperation within the centre (versus with other centres). These secondary analyses can be found in the data files.

### (c) Robustness

In this section, we explore potential methodological limitations and confounds. A first limitation is that formal assessments of participants’ similarity/stereotype perception or group identification were not conducted. However, such an exercise would make the natural identities explicitly salient and there exist ethical reasons to avoid mentioning the natural identity divide during the experiment.

Related to that potential limitation, we analyse the sources of subjects’ perception of within-centre similarity to answer the question, how do the participants compute the likelihood that the counterpart is *G*/*NG*? In particular, we test whether the characteristics of the centre-ingroup members which are correlated with *G*/*NG* status could be used by participants to visually infer the proportion of *G*/*NG* in the room. We test whether the mean age and the proportion of females and/or Spanish participants within each centre sample can predict the proportion of *G* versus *NG* participants in the centre since, as noted above, *G* participants are younger and less likely to be females and Spanish than *NG*. In fact, at the centre-level, the three are significant predictors of *G*/*NG* proportions when introduced individually (all *p* < 0.03; OLS, *n* = 14). However, when all the three variables are introduced at the same time as regressors, the percentage of female participants in the centre is no longer significant (*p* = 0.45), whereas the mean age and the proportion of Spanish participants remain significant (both *p* < 0.01). The latter two variables explain 84% of the variance in the proportion of *G*/*NG* across centres. In other words, participants might have used age and nationality as the main sources for the inference of *G* and *NG* identities. See the analyses in the data files.

In addition, we test whether the effect of centre-ingroup similarity might be reflecting differences in the proportion of *G* versus *NG* individuals in the centre population of users, arising from centre-level omitted variables, rather than among those who finally participated. Since by design of the sampling strategy, the proportion of *G* individuals finally participating within a centre was highly correlated with the proportion of *G* users belonging to the centre population (corr = 0.681; see §4*b*(i)), it might be that the results reflect spurious differences between centres rather than an effect of perceived similarity with the centre-ingroup. To test this alternative, rather than the probability that the partner in the centre sample is *G*/*NG* (our measure of centre-ingroup similarity), we use the proportion of *G*/*NG* users in the same centre population based on the administrative data. The results have similar interpretation, but they are weaker: the larger the relative population of *G* users in the centre, the lower the centre-ingroup favouritism (*p* = 0.045). However, this variable turns non-significant (*p* = 0.70) when we introduce in the regression our sample-level measure (*p* = 0.012). This means that the effect observed for similarity does not seem to be confounded by that of other omitted variables related to the relative proportion of *G*/*NG* users in a centre population. This analysis can be found in the data files.

## 3. Discussion

We study how group diversity interferes with social integration in the context of a mentoring programme. Following the tradition in behavioural economics, we build on a theoretical framework where individual utility depends on both one’s own and the interaction partner’s payoffs [55–57]. Adding identity concerns to individual preferences [50,58], we develop testable predictions without imposing the sign or magnitude of the effect of shared affiliation on cooperation. This framework allows both ingroup and outgroup favouritism for both natural and centre identities.

Our results indicate that the presence of strong natural identities dramatically affects the dynamics of intra- and intergroup cooperation. While *NG* individuals display the ‘standard’ ingroup favouritism when the conditions are closer to those of the typical minimal group paradigm (i.e. as newcomers in the centre) as well as in natural identity, *G* individuals display outgroup favouritism in both cases. Actually, centre-ingroup similarity has a negative effect on centre-ingroup favouritism for *G* individuals and a positive effect for *NG*. Our results have therefore little to say on the debate between social identity and group-bounded generalized reciprocity theories, which mostly neglect outgroup favouritism, and suggest that considerations on system-justification and stereotypes [21,22] should be incorporated. We cannot know if the underlying force behind our results is the expectation of cooperation by others (supporting group-bounded generalized reciprocity [35]), but it is a potential rationale for the stigma effect which would merit further research.

These findings point to a self-fulfilling prophecy [53,54]: centres with a larger number of negatively stereotyped participants show less centre-ingroup (versus outgroup) cooperation, even though on average *G* individuals are not less cooperative. For an external observer, this would potentially reinforce the negative stereotype. Espín *et al*. [3] underlines the role of stereotypes for cooperation processes involving different-status natural identities and are in line with ours: the negatively stereotyped Romani (Spanish *gitanos*) who did not cooperate in ethnically mixed groups were punished more harshly than uncooperative non-Romani (the Spanish majority) by both Romani and non-Romani cooperators, even though the two ethnocultural groups were not differently cooperative on average.

A longer interaction history might increase familiarity and bonds of trust, and *G* individuals with longer stays indeed show *higher* centre-ingroup favouritism. However, *NG* individuals with a longer interaction history show *less* centre-ingroup favouritism. Users in the same centre actually compete for jobs; future research should analyse if this explains the lower centre-ingroup favouritism among more senior *NG* users, which might align with the ‘nasty neighbour’ effect [26]. While we randomly selected users in different stages, self-selection is still a potential confound, however, as it might be that those staying longer in the centre are different. In any case, interaction history is never able to erode the stigma effect of natural identity. The positive effect of interaction history on centre-ingroup favouritism for *G* individuals is consistent with the findings of Balafoutas *et al*. [59], who observe that prison inmates increase their ingroup favouritism (another inmate versus an outgroup from outside prison) the longer they remain incarcerated, as well as with those of Exkel *et al*. [43] in that students show more favouritism towards members of their residential college (versus other colleges) the longer they have interacted within the college.

Contributing to the literature on cooperation in multi-identity contexts, we observe positive additive or synergistic effects of both shared identities [33–35] only for *NG* individuals with little interaction history: for them, centre-ingroup favouritism is positive on average and more positive the more likely the centre-ingroup is also a natural-ingroup. For the other three cases (i.e. *G* individuals with little or long history and for *NG* individuals with long history), the effects of sharing identities are however antagonistic, or even both negative (for little-history *G* individuals). These complexities need to be considered in multi-identity analyses.

Our results may have implications for policy making and for the design of similar programmes. While these programmes might increase the negatively stereotyped individuals’ sense of belonging, they might also lead to a perception of increased competition with their programme mates among other types of users. Further studies should explore the exact factors leading to differential relationships of interaction history with ingroup favouritism for different societal groups.

We hope our work stimulates further empirical research analysing whether social integration is possible under strong group diversity. This is important in increasingly mixed societies. Further research is needed to disentangle the reasons underlying ingroup and outgroup favouritism in the presence of possibly conflicting natural identities, thus complementing leading models on intergroup behaviour and bias [21,60]. Mounting evidence suggests that the outcomes of intergroup contact change along with the type of interaction depending on the entitativity and status of the groups involved, among other factors [3,37,61]. The current findings support such a multifactorial perspective.

## 4. Methods

### (a) Theoretical framework and hypotheses

#### (i) Individual preferences

We consider other-regarding preferences, that is, the individual cares about herself but also about other people and this is reflected in her utility function [55,56], where the payoff of the partner is weighted by a parameter *γ* associated to each interaction partner’s social identity,


U(x,y)=x+γy,

where *x* denotes the individual’s payoff and *y* denotes the partner’s payoff.^3^

Under rational choice, subjects will choose the action that maximizes *U*(*x*, *y*). Three main cases of other-regarding preferences arise [63]: positive regard or altruism (*γ* > 0), negative regard or spitefulness (*γ* < 0), and indifference/self-interest (*γ* = 0).

Our formulation of intergroup behaviour is based on the comparison of the different *γ*’s associated to the groups involved. In each interaction, the individual considers the *γ* of the partner’s social category and, depending on the game structure, favours the members of the group associated to higher *γ*. These model features allow us to test the effects on behaviour of variables such as shared affiliation rather agnostically (i.e. without exogenously imposing their sign and/or magnitude). We focus here on the predictions arising from our model. See electronic supplementary material, §S2 for more theoretical details.

#### (ii) The role of natural and centre identities: predictions

First, we focus on centre identities. The traditional parochial altruism perspective would suggest the existence of centre-ingroup favouritism (prediction A1). Regarding natural identities, we cannot predict *ex ante* whether subjects will cooperate more or less with *G* than with *NG* (they may feel compassion/sympathy for the disadvantaged *G* or they may dislike them). Predictions B1–B3 explore this. In addition, we test if these predictions hold differently depending on the history of interaction within the centre-ingroup.

#### A1. Centre-ingroup favouritism

In this case, centre grouping is enough to yield ingroup favouritism [18]. Individuals have a more positive other-regarding preference (*γ*) towards the centre-ingroup than towards the centre-outgroup, regardless of natural identities. The prediction is: *centre-ingroup cooperation is higher than centre-outgroup cooperation; that is, there is centre-ingroup favouritism on average.*

#### B1. Natural-ingroup favouritism

Here, individuals have more positive other-regarding preferences (*γ*) towards the members of their own versus other natural group [38]. For both natural-identity types, the prediction is that *similarity increases cooperation with the centre-ingroup (versus outgroup), yielding a positive effect of similarity on centre-ingroup favouritism*.

#### B2. Stigma of a natural identity

Here, one of the groups (*G*) has a negative stereotype and is discriminated against [21]. Members of *NG* are unanimously favoured, and all players have more positive other-regarding preferences (*γ*) towards them than towards the stigmatized *G*. The prediction is that *for NG individuals, similarity increases centre-ingroup favouritism; for G individuals, similarity decreases centre-ingroup favouritism.*

#### B3. Compassion/sympathy towards the disadvantaged natural identity

The opposite to stigma implies favouring the disadvantaged *G*, which could arise from compassion or sympathy towards low-status groups [64]. Here, the prediction is that *for NG individuals, similarity decreases centre-ingroup favouritism, whereas for G individuals similarity increases centre-ingroup favouritism.*

#### (iii) Interaction history

One key factor leading to group identification is repeated interaction, probably influenced by the expectation of future interaction [20]. When participants are assigned to different centres based on administrative reasons, their centre can be initially seen as a meaningless group. However, a history of repeated interaction is likely to elicit a stronger sense of belonging and identification, so that the centre becomes a more meaningful group (a ‘weak’ real group becomes a ‘strong’ real group [43]).

With repeated interaction, members of the same centre may be perceived more as individual beings than as members of a particular natural identity [42]. According to this view, there should be an increase in centre-ingroup favouritism (A1) and a decrease in the absolute value of the effect of similarity over time. Since we recruited participants at different stages of the programme, from newcomers to more senior users, we can check whether the centre-group identity becomes more important over time and eventually erodes any effect of natural identities. However, within-centre interaction may result in integration but also in escalation of conflict over time, leading to multiple possible outcomes. Therefore, we test whether the support of our predictions is modulated by interaction history.

### (b) Experimental design

Participants were users of a mentoring programme in Spain focused on young adults (ages between 18 and 35, with an average ± s.d. age of 22.3 ± 4.3). By design, the sample includes participants who were previously in legal guardianship (*G*) as well as other users (*NG*).

#### (i) About the programme

The aim of the mentoring programme is to expand employment opportunities for young vulnerable people at risk of social exclusion in Andalusia, Spain. The programme offers a variety of training activities related to critical skills and competences. The implementation of the programme is carried out through 15 centres. The users enrol voluntarily in the programme activities.

The programme deals with two types of users. Individuals who have been under legal guardianship (*G*) overtly suffer from stigma. They are perceived as more likely to be involved in delinquent activities and other negative stereotypes [10–12]. The rest of participants (*NG*) are not seen as a clearly differentiated group in society.

#### (ii) Sample selection

Our administrative database includes anonymized information about the programme users, with a total of 3533 individuals (18% had been under legal guardianship; 45% females; 73% Spanish; average age = 25).

The experiment was conducted in June and July 2019. To build the sample, we provided the managers of each centre with a list of randomly selected users based on *ex ante* power calculations. Since the main research goal was to compare *G* and *NG* individuals, we set minimum quotas of *G* participants in each centre to increase their share in the sample (which is only 18% in the user database) to approximately 50% while keeping between-centre proportionality (the proportion of *G* individuals in the user population ranged from 3% to 41% across centres). As we expected that not all invited individuals would finally participate, our power calculations were based on a conservative 50% participation rate. The sample size to obtain small-to-moderate differences (Cohen’s *d* = 0.4) between *G* and *NG* individuals with 80% power and alpha = 0.05, two-tailed, was 96 of each type. Thus, we created a list of 384 participants to be invited (192 of each type), selecting individuals in different stages of the programme (both relatively junior and senior users).

This protocol resulted in a sample of 216 participants, with 127 *G* (58.8%) and 89 *NG* individuals (41.2%).^4^ For 14 participants, the date when they entered the programme could not be confirmed. Another seven participants failed to provide an unambiguous response for both PD games. Finally, in one centre only one individual showed up and therefore her PD decision about the centre-ingroup had no real consequences.

After excluding these 22 participants, the final sample consists of 194 individuals (112 *G*, or 57.7%, and 82 *NG*, or 42.3%) from 14 centres, with proportions of *G* ranging from 11% to 100%. The resulting between-centre proportionality regarding the percentage of *G*/*NG* individuals in this final sample versus the population was acceptable (Pearson correlation = 0.681, *p* < 0.01, *n* = 14). This (unbalanced) sample allows to obtain the initial *d* = 0.4 differences between *G* and *NG* with 80% power and alpha = 0.05.

#### (iii) The experiment

The experiment was conducted using paper-and-pencil protocols with 18 pairs of enumerators who read the instructions and navigated participants to complete the responses. All responses were anonymous; enumerators were not allowed to see them.

After a battery of six tasks,^5^ the participants played two consecutive PD games with real stakes [71]. The experimental design was both between and within subjects, as both *G* and *NG* individuals played the game twice: one with an unknown participant from the same centre (centre-ingroup) and another with an unknown participant from a different centre (centre-outgroup). The order was randomized across participants to control for order effects [72].

**Figure 3.**
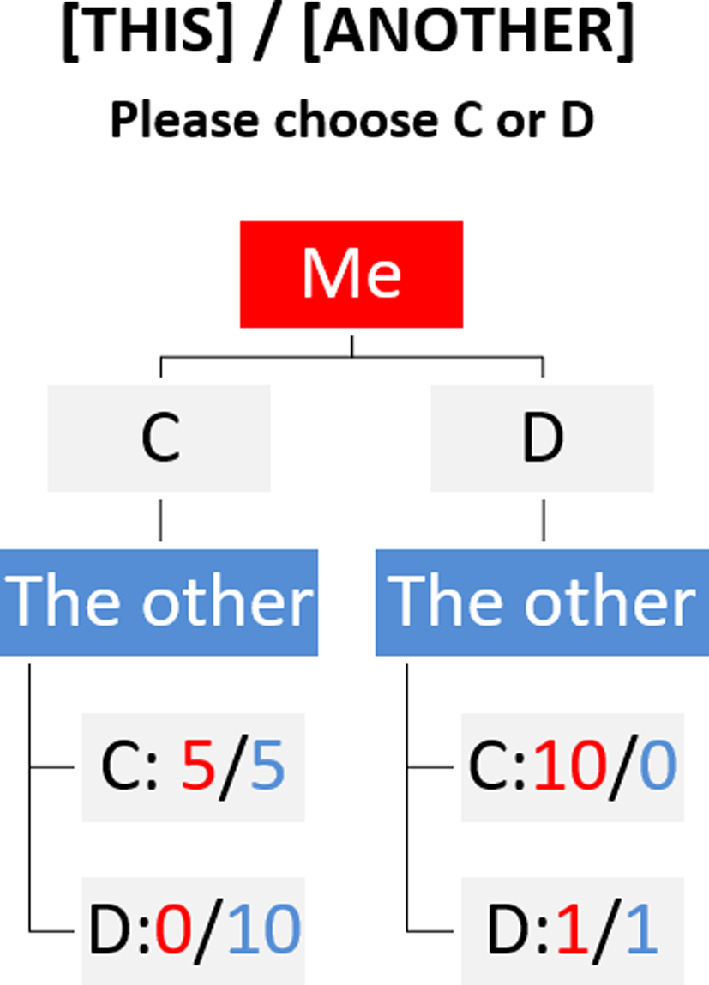
Decision sheet for the PD game with the centre-ingroup (‘THIS’) or centre-outgroup (‘ANOTHER’).

The subjects had to decide whether to cooperate with another participant (binary decision: C or D; see figure 3) in return for experimental points.^6^ They were explicitly informed whether the counterpart belonged to their own or another centre (see electronic supplementary material, §S1 for full instructions).

The *natural identity of the partner* was unknown due to the random matching mechanism. Regarding the centre-ingroup, participants could observe others in the same centre and use that information to compute the probability that their centre-ingroup counterpart was *G* or *NG*. However, they were blind to the natural identities of the centre-outgroup.

Similarity in the experiment is measured by the proportion of participants of the same natural group among the centre participants other than the decision maker. This gives the probability that the centre-ingroup is of the decision maker’s type, ranging from 0 to 1 with an average ± s.d. of 0.611 ± 0.244 in the final sample. Since the programme activities are set weekly, we use the number of weeks the individual had been in the programme before the experiment as our measure of interaction history (average ± SD = 36.36 ± 23.58 weeks; range 0−74). Electronic supplementary material, figure S1, displays the distribution of similarity and interaction history for *G* and *NG* participants.

We have reported all measures, conditions and data exclusions. The sample size was determined through power analysis.

## Data Availability

All the data and code (STATA format) used for this study can be found at [73]. Supplementary material is available online [74].

## Footnotes

^1^
The guardianship may have been set for several reasons: foreigners with no family in the country (unaccompanied minor migrants), native orphans, or kids separated from their families for legal reasons. These are non-standard experimental subjects, a feature that constitutes a contribution of our study in itself. In their systematic review of minimal group studies, Pechar & Kranton [9] indeed identify ‘limitations of the current minimal group literature, including an overrepresentation of findings from white, middle-class, western samples and from experiments with student subjects’.

^2^
Given that participants knew ex-ante that they would make decisions for both centre-ingroups and outgroups, both decisions are necessarily linked. Thus, the most appropriate dependent variable is centre-ingroup favouritism which considers a combined decision strategy. Two additional reasons why centre-ingroup favouritism is the best choice in our case are: (i) Since centre-outgroup cooperation is not affected by similarity in the same centre, the centre-outgroup is a good reference point. (ii) The effect of similarity is considering both natural-identity ingroups and outgroups because it refers to the proportion of *G* and *NG* individuals in the room (i.e. it is ‘comparative’ by definition). Therefore, we need to use ‘comparative’ measures for centre identity.

^3^
More generally, utility could be any increasing function of these two components; for the sake of simplicity, we set a linear functional form in the spirit of Levine [62].

^4^
We could not force the exact 50% proportion but only encourage it with recruiting quotas. We did not have full control over who finally showed up because this was a voluntary activity.

^5^
In particular: (i) self-reported life control, self-esteem and life satisfaction [65]; (ii) questionnaire on self-discipline, impulsivity and assertiveness [65]; (iii) cognitive skills [66]; (iv) self-confidence about the cognitive skills performance [67]; (v) questionnaire on personal life expectations [68]; and (vi) time discounting [69,70].

^6^
The experimental points were exchanged for ‘lottery’ tickets. The more lottery tickets one had, the more likely they were to obtain monetary prizes in a subsequent draw. All the participants’ lottery tickets were introduced into a (virtual) pot and 50 of them were drawn. The draw consisted of 10 prizes of each of the following amounts (€): 100, 50, 20, 10 and 5. Thus, the total amount distributed among the participants was €1850. Just for participation, they received five lottery tickets.
